# Effectiveness of Continuing Education Interventions in Improving Dentists’ Proficiency in Oral Cancer Screening: A Systematic Review and Quality Appraisal

**DOI:** 10.1155/ijod/8479848

**Published:** 2026-10-07

**Authors:** Marina Sulyman, Mariam Demian, Jyothi Tadakamadla, Elizabeth Fitriana Sari

**Affiliations:** ^1^ Dentistry and Oral Health Discipline Department of Rural Clinical Science La Trobe Rural Health School Bendigo VIC Australia; ^2^ Violet Vines Marshman Centre for Rural Health Research La Trobe Rural Health School La Trobe University Bendigo VIC Australia latrobe.edu.au; ^3^ College of Dentistry Clinical Sciences Department Ajman University Jurf 1, J1 Building Ajman UAE ajman.ac.ae

**Keywords:** continuing, dentists, early detection of cancer, education, mass screening, oral cancer

## Abstract

**Background:**

Early detection of oral cancer (OC) is critical for improving survival outcomes; however, delayed diagnosis remains common worldwide. By performing routine OC screenings, dentists play a central role in its early detection yet persistent gaps in knowledge, confidence and clinical skills remain. While previous reviews have focused on diagnostic adjuncts and technologies for OC detection, the effectiveness of continuing educational interventions targeting dentists’ screening proficiency has not been systematically appraised.

**Objective:**

To systematically review and qualitatively appraise the effectiveness of continuing educational interventions in improving dentists’ proficiency in performing OC screenings.

**Method:**

A systematic review and quality appraisal was conducted in accordance with PRISMA 2020 guidelines. Searches were undertaken in MEDLINE, PubMed and CINAHL, supplemented by Google Scholar and citation tracking from inception to January 2026. Eligible studies were English‐language primary research evaluating OC educational interventions for dentists with pre and postintervention outcome assessment. Owing to substantial heterogeneity in study design, intervention characteristics and outcome measures, meta‐analysis was not feasible. A qualitative synthesis was undertaken, and the certainty of evidence was assessed using the GRADE approach. This study has been registered in PROSPERO (REF: CRD420250591937).

**Results:**

Nine studies involving sample sizes ranging from 47 to 442 participants were included, encompassing online, face‐to‐face, blended and campaign‐based educational interventions. All nine studies reported improvements in dentists’ knowledge and awareness following the interventions. Five studies reported improvements in diagnostic accuracy and lesion recognition, five reported increased confidence or self‐efficacy, and three reported improvements in OC screening or referral practices. Most interventions were of short duration and relied predominantly on self‐reported outcomes. Limited undergraduate training, unclear referral pathways and inadequate infrastructure were commonly reported barriers to the implementation of OC screening. The certainty of evidence, assessed using the GRADE approach, ranged from very low to low across the evaluated outcomes.

**Conclusion:**

Continuing educational interventions may enhance dentists’ proficiency in OC screening, particularly when interactive and practice‐oriented methods are used. Nevertheless, integrated approaches combining standardised curricula, longitudinal training and system‐level support are required to translate educational gains into sustained practice change, earlier OC detection and improved outcomes.

## 1. Introduction

Oral cancers (OCs) involve malignancies of the lip and oral cavity, including the tongue, floor of the mouth, buccal mucosa, gingiva, hard and soft palate and oropharynx [1]. OC remains a significant global health burden with persistently high morbidity and mortality rates largely attributable to late‐stage diagnosis [2, 3]. Despite the oral cavity being readily accessible for clinical examination by dentists, a substantial proportion of OCs are still detected at advanced stages, when treatment is more invasive and survival outcomes are poorer [4, 5].

Diagnosing oral cancer (OC) requires thorough examination of the oral mucosa to identify potentially malignant lesions in both high‐ and low‐risk individuals [6, 7]. Dentists also play an important role in referral for specialist assessment and biopsy, as well as patient education and counselling regarding modifiable risk factors [8, 9]. Inadequate screening and operator training have been associated with poorer OC outcomes [10], yet routine screening by dentists remains limited [11, 12].

Several studies have identified substantial gaps in dentists’ knowledge and screening practices. In Indonesia, 63% of dentists were unaware of the early clinical presentation of OC [3], while 95% reported difficulty detecting early premalignant changes because of insufficient undergraduate training [7]. Among Australian dentists, 52.3% and 28.1% were unaware of appropriate oropharyngeal inspection and neck palpation, respectively [5]. In the Democratic Republic of Congo, only 19.8% routinely performed OC screening and 22% reported confidence in providing preventive education [13]. In Malaysia, 75% reported uncertainty regarding referral pathways and 53% were unaware of all OC risk factors [9]. Similarly, a Brazilian study found that ~15% of dentists performed OC screening only when patients reported pain [8].

More than 70% of dentists have demonstrated receptiveness to further OC education and training [8, 9, 13]. Educational interventions implemented across diverse settings, including India, Spain, Saudi Arabia, Northern Germany, Brazil and Indonesia, have reported improvements in dentists’ knowledge, confidence, screening practices and diagnostic accuracy [8, 14–18]. Notably, interventions in Brazil and Indonesia reported increases in diagnostic accuracy of 88.9% and 68.7%, respectively [8, 18]. These findings suggest the potential value of continuing education in addressing gaps in OC screening practice.

Previous systematic reviews on OC have predominantly focused on diagnostic adjuncts, artificial intelligence, imaging modalities and other technologies designed to improve the detection and diagnosis of OC [12, 19]. Although these reviews provide valuable insights into diagnostic performance, they do not specifically evaluate whether continuing educational interventions improve dentists’ knowledge, diagnostic confidence, clinical screening practices, or competency in performing conventional OC examinations. Similarly, reviews of oral health education programmes have largely targeted caries prevention and periodontal disease management, rather than OC screening [20–22]. Furthermore, the effectiveness of continuing professional development (CPD) programs in enhancing dentists’ proficiency in early OC detection has not been comprehensively synthesised or critically appraised.

The need for comprehensive educational strategies that integrate knowledge of risk factors, clinical examination techniques and referral pathways to improve dentists’ screening performance has been alerted to [23, 24]. In addition, concerns have been raised regarding inconsistencies in OC education at the undergraduate level, with evidence suggesting variable preparedness among dental graduates [24]. Collectively, these findings underscore the importance of continuing education as a mechanism for addressing both knowledge and skills gaps among practicing dentists.

Therefore, the aim of this systematic review and quality appraisal is to evaluate the effectiveness of continuing educational interventions in improving dentists’ proficiency in performing OC screenings. Specifically, this review sought to assess whether such interventions improve the knowledge, diagnostic accuracy, confidence and screening practices amongst dentists, identify the effective and ineffective components of existing programs, explore barriers limiting implementation and highlight directions for future research for the development of OC CPD courses and its integration in undergraduate dental degrees.

## 2. Methods

### 2.1. Protocol and Reporting Guidelines

This systematic review was conducted and reported in accordance with the Preferred Reporting Items for Systematic Reviews and Meta‐Analyses (PRISMA) 2020 statement. The review protocol was prospectively registered with the International Prospective Register of Systematic Reviews (PROSPERO; Registration No. CRD420250591937) before the commencement of the review.

### 2.2. Eligibility Criteria

The review question was developed using the population–intervention–outcome (PIO) framework. Eligible studies included qualified dentists, dental practitioners, dental interns, or recent dental graduates who participated in continuing educational interventions related to OC screening, early detection, diagnosis, or management of oral potentially malignant disorders (OPMDs).

The educational interventions included face‐to‐face workshops, online learning, blended learning, CPD programmes, educational campaigns, seminars and structured training programmes.

Studies were eligible if they evaluated changes in one or more of the following outcomes, including knowledge of OC or OPMDs, diagnostic accuracy or clinical examination skills, confidence or self‐efficacy, screening or referral practices, or patient‐related outcomes. Eligible primary research studies included pre–post intervention, quasi‐experimental, controlled intervention, cohort and mixed‐methods designs.

Studies were excluded if they focused exclusively on undergraduate dental curricula without continuing education, evaluated diagnostic technologies without an educational intervention, were reviews, editorials, conference abstracts, case reports, or protocols, or were not published in English.

### 2.3. Information Sources and Search Strategy

A comprehensive electronic literature search was conducted in MEDLINE (via Ovid), PubMed and CINAHL from database inception to January 2026.

Although PubMed includes MEDLINE‐indexed records, it also contains publisher‐supplied citations, ahead‐of‐print articles, and records that are not yet indexed in MEDLINE. Therefore, both MEDLINE and PubMed were searched to maximise the retrieval of relevant studies.

To identify additional eligible studies, backward and forward citation searching of the included articles and relevant systematic reviews was also performed manually.

The search strategy combined medical subject headings (MeSH) and free‐text terms related to dentists, OC, continuing education, OC screening and diagnostic competence, using Boolean operators (AND/OR). Database‐specific search strategies were adapted as appropriate for each database.

The complete search strategies for all databases are provided in Table S1.

### 2.4. Study Selection

All retrieved records were imported into EndNote 21 (Clarivate Analytics, Philadelphia, PA, USA) for duplicate removal before being exported to Covidence (Veritas Health Innovation, Melbourne, Australia) for study screening.

Two reviewers independently screened the titles and abstracts of all retrieved records against the predefined eligibility criteria. Full‐text articles considered potentially eligible by either reviewer were subsequently assessed independently for inclusion.

Any disagreements arising during the title/abstract screening or full‐text review were resolved through discussion until consensus was reached. Where consensus could not be achieved, a third reviewer was available to adjudicate.

The study selection process is presented in the PRISMA 2020 flow diagram (Figure 1).

**Figure 1:**
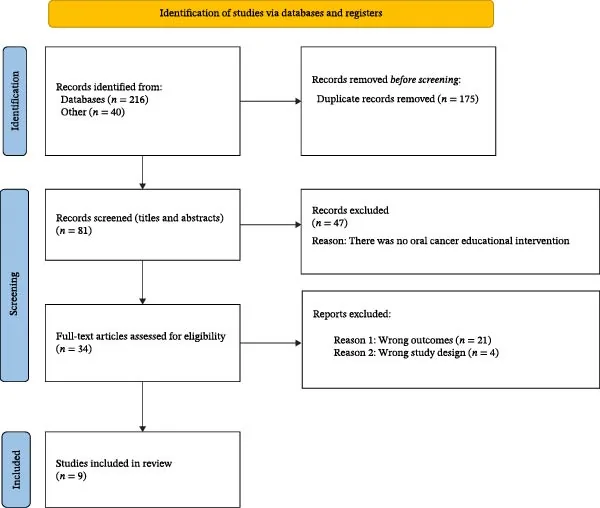
A flow chart of the selection process according to PRISMA 2020.

### 2.5. Data Extraction

A standardised data extraction form was developed and piloted before use. Two reviewers independently extracted data from each included study. Extracted information including author and publication year, country, study design, participant characteristics, intervention characteristics, duration of intervention, follow‐up period, outcome measures, principal findings and study limitations.

Disagreements were resolved through discussion until consensus was achieved. No assumptions were made regarding the missing data.

### 2.6. Outcomes

The primary outcome was the effectiveness of continuing educational interventions in improving dentists’ competence in the OC screening. Secondary outcomes include knowledge of OC and OPMDs, diagnostic accuracy, confidence or self‐efficacy, OC screening practices and referral behaviour.

### 2.7. Certainty of Evidence

Given the substantial clinical and methodological heterogeneity among the included studies, including differences in participant populations, educational interventions, outcome measures and follow‐up periods, a quantitative meta‐analysis was not considered appropriate. Instead, a structured narrative synthesis was undertaken.

To evaluate the overall strength and certainty of the available evidence across the principal outcome domains, the Grading of Recommendations Assessment, Development and Evaluation (GRADE) framework was applied [25, 26]. GRADE was selected because it provides a transparent and systematic approach for assessing the certainty of evidence across a body of literature, even when findings are synthesised narratively. The assessment considered five domains: risk of bias, inconsistency, indirectness, imprecision and publication bias. Based on these domains, the certainty of evidence for each outcome was classified as high, moderate, low, or very low.

Because this review did not aim to generate pooled effect estimates and the included studies were predominantly uncontrolled pre–post intervention studies, the certainty assessment focused on the overall body of evidence for each outcome rather than on individual study‐level quality scores.

### 2.8. Data Synthesis

Owing to substantial heterogeneity in study design, participant characteristics, educational interventions, duration of follow‐up and outcome measures, statistical pooling of results was not appropriate. Consequently, findings were synthesised narratively in accordance with guidance for systematic reviews where meta‐analysis is not feasible.

Studies were grouped according to the principal educational outcomes evaluated, including knowledge and awareness of OC and OPMDs; diagnostic accuracy and lesion recognition; confidence and self‐efficacy; OC screening and referral practices; and patient‐related outcomes where reported.

Quantitative findings reported by individual studies, including changes in knowledge scores, diagnostic accuracy, confidence and screening practices, were summarised descriptively without calculation of pooled effect estimates.

### 2.9. Reporting Bias

A formal statistical assessment of reporting or publication bias was not undertaken because no meta‐analysis was performed and fewer than 10 studies met the eligibility criteria. Potential reporting bias was considered qualitatively during the interpretation of the evidence.

### 2.10. Effect Measures

Due to heterogeneity, no quantitative effect measures were applied; findings were synthesised narratively.

### 2.11. Sensitivity Analysis

Sensitivity analyses were not performed due to qualitative synthesis.

### 2.12. Protocol Access

The protocol is registered in PROSPERO (CRD420250591937); the full protocol is available upon request.

### 2.13. Data Handling Methods

No data transformation was required as synthesis was narrative.

## 3. Results

### 3.1. Overview of the Search Process

A total of 216 articles were retrieved from the database searches (Medline, PubMed and CINAHL) and 40 articles from Google Scholar and citation tracking. All the citations of the 256 articles were imported into EndNote 21, where 175 duplicates were removed. The citations of the remaining 81 articles were imported into Covidence for the initial screening of titles and abstracts against the eligibility criteria and 47 articles were removed as they had no OC educational intervention.

The second screening included 34 articles, where their full texts were assessed, and 21 articles were removed as they had wrong outcomes and 4 were the wrong study design. Nine studies were included in the data extraction process.

Following data extraction of the nine articles, there was substantial heterogeneity amongst the study designs, interventions, outcome measures and mixed‐method evidence; thus, a meta‐analysis could not be performed. As such, a systematic review and quality appraisal was undertaken to critically evaluate the methodological quality, credibility and relevance of the nine included studies as the data could not be statistically pooled. A detailed mapping of study characteristics and outcomes are provided in Table 1.

**Table 1:** Evidence map and descriptive appraisal of oral cancer education interventions.

Study (Country)	Study design and participants	Educational intervention	Outcome measures	Main findings	Follow‐up/limitations
Seoane‐Lestón et al. (2010) Spain [17]	Uncontrolled pre–post study; 440 dentists	4‐h CPD workshop delivered as part of a national oral cancer campaign	Knowledge, diagnostic accuracy	Higher postintervention knowledge and improved recognition of oral lesions	Immediate assessment only; no long‐term follow‐up
Hertrampf et al. (2011) Germany [27]	Uncontrolled pre–post study; 394 dentists	National educational campaign using brochures, posters and DVDs	Knowledge	Higher postintervention knowledge following the campaign	Passive educational approach; self‐reported outcomes
Hertrampf et al. (2013) Germany [16]	Uncontrolled pre–post study; 394 dentists	Educational package (brochures, DVDs and campaign materials)	Knowledge	Higher postintervention knowledge of oral cancer and OPMDs	No patient‐level outcomes; limited participant engagement
Braun et al. (2021) Brazil [8]	Uncontrolled pre–post study; 221 dentists	4‐h ‘Maio Vermelho educational campaign	Knowledge, confidence, lesion recognition	Higher postintervention confidence and lesion recognition	Short intervention; no long‐term follow‐up
Nethan et al. (2022) India [14]	Uncontrolled pre–post study; 52 dentists	14‐week online curriculum followed by 1‐day workshop	Knowledge, confidence, screening practice	Higher postintervention knowledge, confidence and reported screening practice	Participant attrition during follow‐up
Moafa et al. (2022) Saudi Arabia [15]	Mixed‐methods pre–post study; 47 dental interns	6‐month ISAC programme incorporating role‐modelling and communication training	Knowledge, confidence, communication skills	Higher postintervention confidence and communication skills	Small sample size
Gaballah et al. (2022) United Arab Emirates [28]	Uncontrolled pre–post study; 67 participants	4‐h workshop using lectures, problem‐based learning and quizzes	Knowledge, diagnostic accuracy	Higher recognition of benign lesions and OPMDs following the intervention	Limited improvement in malignant lesion recognition; participant attrition
Cardoso et al. (2022) Brazil [2]	Uncontrolled pre–post study; 442 dentists	3‐month distance‐learning programme using PBL and flipped classroom	Diagnostic accuracy, referral practice	Higher lesion classification accuracy and fewer unnecessary referrals	No long‐term clinical follow‐up
Sari et al. (2023) Indonesia [18]	Uncontrolled pre–post study; 323 dentists	1‐day education, 1–2‐day primary healthcare hands‐on training and follow‐up	Knowledge, confidence, lesion recognition	Higher postintervention knowledge, confidence and recognition of oral cancer and OPMDs	Follow‐up at 6–8 weeks; reported contextual barriers to implementation

### 3.2. Study Characteristics

The characteristics of the included studies are presented in Table 1. The nine included studies were published across a range of geographical contexts, including India [14], the United Arab Emirates [28], Saudi Arabia [15], Northern Germany [16, 27], Spain [17], Brazil [2, 8] and Indonesia [18]. Study populations included dental interns and recent graduates [15, 28] to practicing dental practitioners [2, 8, 14, 16, 17, 27] and multiprovince national cohorts [18] with sample sizes varying from 47 to 442 participants. Younger dentists and interns often displayed enthusiasm for training [15, 28] practicing general dentists frequently report insufficient confidence and preparedness [2, 16, 18].

Educational interventions varied considerably in design, duration and mode of delivery. Formats included fully online courses, face‐to‐face workshops, blended learning approaches, national awareness campaigns and problem‐based or flipped classroom models. Intervention duration ranged from single‐day workshops [8, 17, 18, 28] to longitudinal programs extending up to 1 year [16, 27].

### 3.3. Effects of Educational Interventions on Knowledge and Awareness

All nine included studies reported improvements in dentists’ knowledge and awareness following participation in OC educational interventions. Knowledge gains were observed across a range of intervention formats and durations, with the most pronounced improvements reported in studies incorporating structured curricula and interactive components such as blended learning, problem‐based learning and longitudinal delivery [2, 14, 15].

Baseline knowledge levels varied considerably between studies. In large‐scale national campaigns and population‐level interventions, baseline knowledge was often relatively high, thereby limiting the magnitude of postintervention change [27]. Intervention relying primarily on passive educational materials, including brochures and DVDs [16, 27] produced more modest gains in knowledge compared with interactive and skills‐based formats [2, 14, 15].

### 3.4. Effects on Diagnostic Accuracy and Clinical Skills

Studies evaluating diagnostic accuracy reported increases in lesion recognition following educational interventions, although the reported outcomes varied across studies because of differences in educational interventions, assessment methods, participant populations and outcome measures [2, 8, 17, 18, 27, 28]. Several studies also reported changes in referral practices following the intervention [2, 8, 17, 18, 27, 28]. Interactive workshops and case‐based educational sessions reported higher postintervention recognition of benign lesions and OPMDs [28], whereas distance‐learning programmes incorporating diagnostic reasoning and clinical case simulations reported higher lesion classification accuracy together with fewer unnecessary referrals [2].

Improvements in the recognition of malignant lesions were reported less consistently across the included studies. Studies evaluating short‐duration educational interventions or single‐session workshops generally reported little or no significant improvement in malignant lesion detection during the reported follow‐up period [8, 17, 27, 28].

### 3.5. Effects on Confidence and Self‐Efficacy

Six studies reported significant improvements in dentists’ self‐reported confidence and self‐efficacy following educational interventions [2, 8, 14, 15, 17, 18]. Participants commonly described increased confidence in performing comprehensive oral examinations, recognising suspicious lesions and initiating referrals. Interventions combining theoretical instruction with practical or case‐based components were particularly effective in enhancing confidence [14, 15, 18].

Confidence was measured using self‐reported questionnaires administered before and after the educational intervention. Follow‐up duration differed between studies. Only two studies incorporated follow‐up assessments to evaluate the durability of confidence gains over time [14, 15].

### 3.6. Contextual Diversity

Studies conducted in high‐income countries (HICs), including Brazil [2], Northern Germany [16, 27] and Spain [8, 17], evaluated educational interventions delivered through face‐to‐face, online, or blended learning approaches. Several studies were implemented in collaboration with national awareness campaigns or professional organisations and generally included larger participant samples. The Brazilian study [2] evaluated a distance‐learning programme designed to provide continuing education across geographically dispersed regions.

Studies conducted in low‐ and middle‐income countries (LMICs), including India [14, 15] and Indonesia [18], also evaluated educational interventions using a range of delivery methods. These studies generally included smaller participant samples and reported participant attrition and challenges related to educational infrastructure and access in geographically remote areas. The Indonesian study [18] contributed evidence from Southeast Asia, expanding the geographical representation of the included studies.

### 3.7. Practice Integration and System‐Level Barriers

Evidence regarding the integration of screening practices into routine clinical care was limited. Two studies reported increased intention to perform screenings or self‐reported changes in practice [14, 16]. Structural and systemic barriers emerged as recurring themes, including limited infrastructure, lack of referral clarity and insufficient undergraduate OC education and training [8, 18, 28]. One study [14] discussed how workplaces lacked resources to perform OC screenings. Another study [18] provided particularly strong evidence of system‐level constraints, reporting that a majority of dentists did not routinely perform OC screens due to the lack of support systems from healthy policies and uncertainty regarding referral pathways.

### 3.8. Gaps Identified Across Studies

Several common findings were reported across the nine included studies. Three studies [8, 18, 28] reported limited undergraduate education in OC and OPMDs. One study [18] reported that more than 90% of participating dentists perceived their undergraduate training in OC as inadequate.

Most educational interventions were of short duration and evaluated outcomes immediately after the intervention or within a limited follow‐up period [8, 17, 27, 28]. Two studies included longer‐term follow‐up assessments, although participant attrition was reported [14, 15].

Two studies reported challenges in applying knowledge and skills in clinical practice following the educational intervention, including limited clinical resources [14] and referral pathways [18]. Studies conducted in HICs [2, 16–18] primarily described continuing education programmes delivered within established educational systems, whereas studies conducted in LMICs [14, 15, 18] also reported contextual factors related to educational infrastructure, access to continuing education and healthcare service delivery.

The included studies evaluated a range of educational approaches, including interactive workshops, problem‐based learning, online learning, blended learning and passive educational resources such as brochures and DVDs [2, 14, 16, 27, 28].

### 3.9. Strengths and Limitations

Reported strengths included high attendance and satisfaction [8, 14, 15, 17], nationwide reach and scalability [2, 16, 27], demonstrable knowledge and confidence gains [2, 8, 14, 15, 17, 28], and the value of interactive or blended methods [2, 15]. One study [18] added evidence of widespread recognition among practitioners of the importance of OC education, with respondents expressing willingness to expand their role if better trained and supported. The synthesis of evidence across diverse educational formats and healthcare contexts allowed comparison of intervention characteristics and their influence of screening‐related outcomes.

Limitations included participant attrition and restricted sampling [14, 17, 28], reliance on passive materials such as brochures or DVDs [16], short intervention duration limiting sustained impact [8, 17, 28], and limited assessment of long‐term outcomes across most studies. Systemic shortcomings such as insufficient undergraduate training, low screening frequency and uncertainty around referral pathways identified [18] indicated that educational gaps are compounded by structural barriers within health systems.

### 3.10. GRADE Assessment

The certainty of evidence for each outcome was assessed using the GRADE approach, considering study limitations, inconsistency, indirectness, imprecision and publication bias [25, 26]. Overall, the certainty of evidence ranged from very low to low across outcomes (Table 2).

**Table 2:** GRADE evidence profile – oral cancer education interventions.

Outcome	Contributing studies	Summary of findings	Risk of bias	Inconsistency	Indirectness	Imprecision	Publication bias	Overall certainty (Grade)
Knowledge and awareness of oral cancer and OPMDs	Seoane‐Lestón et al. [17]; Hertrampf et al. [27]; Hertrampf et al. [16]; Nethan et al. [14]; Moafa et al. [15]; Gaballah et al. [28]; Braun et al. [8]; Cardoso et al. [2]; Sari et al. [18]	All nine studies reported higher postintervention knowledge or awareness compared with baseline. However, the educational interventions, assessment methods, and outcome measures varied across studies.	Serious	Moderate	Not serious	Serious	Suspected	Low
Diagnostic accuracy and lesion recognition	Seoane‐Lestón et al. [17]; Gaballah et al. [28]; Braun et al. [8]; Cardoso et al. [2]; Sari et al. [18]	Five studies reported higher postintervention diagnostic accuracy or lesion recognition. Improvements in recognising benign lesions and OPMDs were more consistently reported than improvements in recognising malignant lesions.	Serious	Moderate	Not serious	Serious	Suspected	Low
Confidence and self‐efficacy	Nethan et al. [14]; Moafa et al. [15]; Gaballah et al. [28]; Braun et al. [8]; Sari et al. [18]	Five studies reported higher confidence or self‐efficacy related to oral cancer screening, lesion recognition, patient counselling, or referral following the educational intervention.	Serious	Moderate	Not serious	Serious	Suspected	Low
Screening and referral practices	Nethan et al. [14]; Cardoso et al. [2]; Sari et al. [18]	Three studies reported changes in oral cancer screening practices, clinical examination, or referral behaviour following educational interventions. These outcomes were primarily based on self‐reported practice measures.	Serious	Serious	Moderate	Serious	Suspected	Very low
Patient‐related outcomes	None	None of the included studies evaluated patient‐centred outcomes such as earlier diagnosis, stage at diagnosis, treatment outcomes, or survival following educational interventions.	—	—	—	—	—	Not assessed

The certainty of evidence was downgraded primarily because of methodological limitations, including the predominance of uncontrolled pre–post study designs, reliance on self‐reported outcomes, short intervention and follow‐up periods, imprecision associated with small sample sizes, and heterogeneity in educational interventions and outcome measures. The highest certainty of evidence was observed in studies incorporating validated assessment tools, hands‐on components and follow‐up evaluations [2, 14, 18].

### 3.11. Methodological Characteristics

All included studies employed uncontrolled pre–post intervention designs. No randomised controlled trials were identified. The educational interventions varied substantially with respect to participant populations, intervention content, duration, outcome measures and follow‐up periods. This heterogeneity precluded quantitative synthesis and meta‐analysis.

## 4. Discussion

### 4.1. Principle Findings in Relation to Review Aims

This systematic review and quality appraisal synthesised evidence from nine studies evaluating continuing OC educational interventions designed to improve dentists’ proficiency in performing OC screenings.

Collectively, as outlined in Table 3, the findings indicate that these interventions may improve dentists’ knowledge, diagnostic confidence, and self‐reported screening practices. These outcomes align with broader evidence demonstrating that continuing educational activities are a valuable strategy for enhancing OC knowledge, engagement and professional self‐confidence among dental practitioners [29–31].

**Table 3:** Opinion and future research recommendations.

Journal	Opinions to discuss	Future research recommendations
Nethan et al. [14]	Participants wanted hands‐on workshops. Confidence improved after training, and screenings increased (though <50 patients/week, possibly due to patient flow). Courses were considered beneficial and recommended. Training programmes allowed for questions, clarifications and repetition. Workplaces were often unequipped for screenings.	Training programmes should not only increase knowledge but also support translation into practice. Need for hands‐on components and better scheduling. Dentists must be equipped with skills for screening and cessation advice. Develop supportive tools to make screening easier in practice.
Moafa et al. [15]	Participants requested integration of information into dental software or mobile apps, especially for those working alone in rural areas. Many valued the intervention but wanted it linked to continuous professional education and dental degrees.	Explore use of online tools, apps, or clinical software for chairside support. Strengthen oral cancer screening content in dental curricula, including biopsies and patient communication skills.
Hertrampf et al. [27]	Feedback indicated the need to consider different approaches to oral cancer interventions to ensure broader engagement and impact.	Consider innovative and varied approaches in intervention design to improve uptake and effectiveness.
Gaballah et. Al. [28]	Participants highlighted insufficient time spent on oral cancer practical training in undergraduate curricula. Current training does not adequately prepare dentists for practice.	Improve undergraduate oral cancer education by increasing time and emphasis on practical components. Evaluate the effectiveness of existing curricula.
Cardoso et al. [2]	Distance learning produced knowledge gains and improved recognition of premalignant/malignant lesions. Participants felt more confident diagnosing and referring cases, helping reduce public waitlists. Course reach across all of Brazil reinforced the utility of online formats.	Develop online oral cancer courses (with mobile aids) to expand access for rural and remote dentists. Ensure modules address recognition and diagnosis skills.
Hertrampf et al. [16]	Regular oral cancer education courses improved dentists’ knowledge, skills and confidence. Repeated interventions were seen as necessary for reinforcement.	Establish regular oral cancer education courses as part of continuous professional development.
Seoane‐Leston et al. [17]	While improvements were seen, it was stressed that ongoing educational efforts are mandatory to maintain competence in full‐mouth examinations. Suggested creating international networks and accessible online resources to support training.	Continue implementing continuing education and develop a global online platform (like a ‘trauma guide’) for oral cancer training. Ensure it is free and user‐friendly to reach rural, remote and metropolitan dentists alike.
Braun et al. [8]	Need for more oral medicine/oral pathology practical training in undergraduate programmes. Practical skills risk being lost if not reinforced. Active engagement in screening improves long‐term self‐efficacy. Curriculum inconsistency worldwide raises the need for debate on minimum standards.	Improve undergraduate curricula with more time and practical training in oral cancer. Standardise curriculum globally and define the minimum number of hours for effective training.
Sari et al. [18]	GDPs overwhelmingly reported insufficient training (92.7%), with >50% not screening or educating patients and 22.6% not referring suspicious lesions. Many lacked awareness of referral pathways, particularly in underserved provinces. These findings reflect systemic inequities in training and infrastructure, beyond individual practice gaps.	Conduct longitudinal and intervention studies to evaluate whether structured training translates into sustained practice. Reform undergraduate curricula to include more practical OC/OPMDs components. Develop referral networks and supportive infrastructure. Explore cost‐effective national training strategies and policy‐level solutions.

However, the magnitude and consistency of reported improvements varied according to the intervention design and delivery format. Interventions incorporating interactive elements, blended delivery and longitudinal approaches were more frequently associated with favourable outcomes, whereas short‐duration or predominately passive formats (e.g., brochures DVDs, or single lectures) were associated with more limited reported benefits.

Notably, improvements were more consistent for knowledge acquisition and confidence than for diagnostic accuracy in identifying malignant lesions. The need for enhanced diagnostic accuracy is well‐recognised with studies emphasising that dentists require additional training, reinforcement and adjunctive tools to improve consistency and reliability in early detection and referral decisions [31, 32].

### 4.2. Education Design and Learning Outcomes

Overall, the synthesis suggests that effective OC education for dentists may benefit from interactive, practice‐orientated and longitudinal learning models. Hands‐on workshops, problem‐based learning and blended delivery formats appear potentially valuable for developing diagnostic reasoning and clinical confidence by allowing participants to engage in clinical reasoning, lesion recognition and case‐based decision‐making [29, 31]. In contrast, passive educational approaches were associated with more limited and short‐lived reported improvements, reinforcing the need to move beyond information dissemination and towards interactive, skills‐based and behaviourally anchored learning models [33].

This distinction is particularly important in the context of OC screening, where diagnostic accuracy depends not only on factual knowledge but also on visual recognition, pattern differentiation and decision‐making under uncertainty [33]. Accordingly, the findings suggest potential value in complementing knowledge‐based educational metrics with competency‐based and performance‐linked training models [30]. Sustained training and repeated exposure may be beneficial in supporting long‐term retention of diagnostic skills and improve recognition of malignant lesions.

Digital and distance‐learning platforms may offer scalability and improved access, particularly for practitioners in rural or underserved areas, and were widely endorsed in several studies due to shortages in continuing education opportunities in underserved and rural areas [30]. However, digital or online learning may be more beneficial when complemented by opportunities for practical skill development.

### 4.3. Interpretation in Relation to Existing Literature

The findings of this review complement and extend the existing literature that has largely focused on diagnostic adjuncts and screening technologies for OC detection. While adjunctive tools such as autofluorescence, AI‐based technology, vital staining, and digital imaging have been reported to improve diagnostic accuracy, their effectiveness may be influenced by operator training, baseline knowledge, infrastructure and system‐level support [31, 34]. This synthesis underscores that competence in clinical examination, risk assessment and diagnostic reasoning remains fundamental to effective OC screening, and that continuing education for dentists represents a necessary prerequisite for the successful integration of diagnostic innovations.

As discussed in three of the included studies [8, 18, 28], there is evidence of insufficient training in undergraduate dental programmes, consistent with prior reports, identifying substantial variability and insufficiency in undergraduate OC education. Several studies have highlighted inadequate baseline preparation among dentists and recent graduates. Key barriers included limited knowledge on risk factors, difficulty identifying at‐risk patients, lack of confidence with performing OC screenings and patient communication, and insufficient understanding of oral mucosal changes, all of that may contribute to reduced screening practices [1, 5, 35, 36]. International evidence indicates that OC education is often minimally addressed in dental curricula, leaving dental practitioners underprepared to perform OC screenings and detection and provide patient education [7, 29, 36].

As demonstrated in two studies [16, 27], passive and short‐duration educational interventions were associated with limited reported improvements, suggesting that current OC curricula and continuing educational courses may not adequately support sustained learning or behavioural change. These findings suggest potential value in adopting a more rigorous longitudinal format incorporating repeated exposure, active learning and contextual application to achieve sustainable clinical competence [24, 37].

Standardisation of OC education within undergraduate curricula with a minimum agreed contact hours with clearly defined competency outcomes and integrating different delivery formats may strengthen the current education framework. Practical and longitudinal CPD embedded within national health education frameworks and aligned with public health policies may support sustainability and long‐term impact [24, 30]. Collectively, these findings suggest a need to consider a standardised, outcome‐linked continuing education framework to improve early OC detection in general dental practice.

### 4.4. System‐Level and Contextual Considerations on Practice Translation

A key contribution of this review is the identification of systemic and contextual barriers that may limit the translation of educational gains into routine practice. Evidence from low‐ and middle‐income country (LMIC) settings, highlighted pervasive deficiencies in undergraduate curricula, unclear referral pathways, limited access to specialist services and inadequate clinical infrastructure [18, 34, 35, 38]. In particular, studies reported that the lack of facilities, funding, and infrastructure represents one of the greatest challenges to early OC detection, often outweighing individual level knowledge gains [38]. Educational interventions alone may therefore be insufficient to produce sustained improvements in screening behaviour without concurrent system‐level support.

Organisational barriers such as remote and inaccessible healthcare facilities, lack of transportation, long waiting times and ineffective referral systems were consistently identified as contributors to delayed diagnosis, particularly in rural and underserved regions [35]. These findings are supported by global data indicating that primary healthcare centres in LMICs frequently lack basic equipment for oral inspection and digital documentation, further limiting the practical application of screening skills acquired through education [34]. Workforce shortages, uneven distribution of dental services, longer travel distances and financial barriers further compound delays in diagnoses [35]. Importantly, these barriers are not solely resource‐related but are also linked to governance and policy gaps, including the absence of standardised OC screening guidelines and national referral pathways [39].

In contrast, studies conducted in high‐income country (HIC) contexts often benefited from established professional networks and national campaigns, facilitating broader dissemination and reach. Countries such as Australia, Denmark, Sweden, Norway, the UK and Japan have implemented national cancer care pathways to reduce waiting times and improve timeliness in care [39]. However, even in HIC settings, limited follow‐up and short intervention duration remained persistent challenges, underscoring the need for sustained and international system‐integrated educational approaches [39].

### 4.5. Clinical and Policy Implications

From a clinical perspective, improving dentists’ proficiency in OC screening requires supportive practice environments that enable application of acquired skills. Clear referral pathways, access to specialist services, and integration of screening protocols into routine dental care may facilitate the translation of educational gains into clinical practice [32, 40].

At the policy level, coordinated collaboration between academic institutions, professional bodies and health authorities is required to align educational initiatives with national cancer control strategies. International organisations such as the World Health Organisations, American Society of Clinical Oncology and Institute of Medicine have highlighted the potential of standardised care pathways to reduce variation, enhance accessibility, and improve quality and cost‐effectiveness of care [39]. Policy reforms addressing funding, infrastructure and insurance coverage are particularly critical in LMICs, where financial barriers and late‐stage diagnosis remains disproportionately high [34, 35, 41].

The exploration of digital and mobile tools for chairside decision support, teleconsultation and smartphone‐enabled telemonitoring represents a promising strategy to extend specialist input to rural and resource‐limited settings [12]. Given the limited availability of specialists in rural and remote areas, initial consultations via telemonitoring may reduce geographic and financial barriers to accessing care [34]. Evidence suggests that such approaches can achieve high diagnostic concordance with in‐person assessments when implemented within standardised protocols [34]. Further government support is required to expand specialist availability in out‐reached areas and strengthen referral pathways for improving early OC diagnosis [35]. Building on international cooperation networks and digital platforms for global knowledge sharing supported by system‐level infrastructure, including access to digital photography, and standardised referral templates, may further enhance screening capacity [34, 40].

Overall, this review directly addresses its primary aim of evaluating the effectiveness of continuing educational interventions to better improve dentists’ proficiency in OC screening, while also identifying contextual, systemic and policy‐related factors that influence the translation of educational gains into routine clinical practice [42]. The findings demonstrate that continuing education may enhance dentists’ knowledge, diagnostic confidence, and screening‐related behaviours; however, they also indicate that education alone is insufficient to achieve sustained improvements in early OC detection without concurrent organisational and policy support [42].

From both clinical and policy perspectives, the findings highlight that the impact of educational interventions is context‐dependent, with greater effectiveness observed when training is embedded within coordinated and well‐resourced healthcare systems [34, 42]. These insights are directly aligned with the review’s stated aims of informing education design, clinical implementation, and policy development to strengthen early OC detection across diverse healthcare settings.

### 4.6. Limitations of the Review

Several limitations must be acknowledged. Substantial heterogeneity in intervention formats, outcome measures and follow‐up durations precluded quantitative synthesis and limited cross‐study comparability. Most included studies relied on self‐reported outcomes, introducing potential social desirability and recall bias. Short follow‐up periods further restricted assessment of sustained behaviour and policy change. While all studies employed within‐group pre and postintervention designs, the absence of control groups limited causal inference and increased susceptibility to confounding factors.

### 4.7. Future Research Priorities

As outlined in Table 3, participants consistently valued training programmes but identified unmet needs. These included requests for hands‐on workshops [14], integration of clinical tools into software or mobile apps [15], and stronger emphasis on practical skills in undergraduate curricula [8, 28]. Distance learning was positively received for extending access to rural practitioners [2], while national campaigns reinforced the importance of continuing education [16, 17]. One study [18] amplified these perspectives by documenting practitioners’ frustration with inadequate training and infrastructure, and their call for clearer referral pathways.

As such, several areas should be prioritised for future research as summarised in Table 3.

First, developing practical, hands‐on, and longitudinal training programmes explicitly linked to clinical implementation and behavioural change [14, 16, 18, 28].

Second, exploring digital and mobile technologies as chairside decision‐support tools to broaden reach, scalability and clinical decision‐making [2, 15]. Emerging digital platforms, mobile applications, and artificial intelligence–assisted resources may enhance risk assessment, lesion recognition and referral decision‐making in general dental practice.

Third, evaluating innovative and inclusive educational approaches designed to broaden reach and engagement across diverse dental settings [27]. Flexible delivery models, including blended, asynchronous and technology‐enhanced learning formats, may help overcome barriers related to time constraints, geographic location and workforce diversity.

Fourth, standardising OC undergraduate curricula, including minimum agreed teaching hours and core competency outcomes [8, 18, 28]. Greater curricular consistency would improve educational quality assurance, facilitate cross‐study comparison and support workforce‐wide competence in OC screening.

Fifth, building international collaborative networks and shared digital platforms for support global knowledge exchange, harmonisation of OC educational standards, and capacity building across different healthcare contexts [17].

Finally, future moving beyond individual‐level education and assessing system‐level interventions, including referral pathways, infrastructure support and alignment with health policy frameworks [18]. Educational interventions are most likely to translate into improved patient outcomes when embedded within supportive organisational and policy environments. A study explains how without understanding the root causes of inequities, resource allocation and the need for policy change, strengthening oral health service access, availability and affordability to LMICs would be unachievable [42].

## 5. Conclusion

This systematic review suggests that continuing educational interventions may improve dentists’ knowledge, diagnostic confidence, and OC screening practices. However, the available evidence is derived predominantly from uncontrolled pre–post studies and was judged to be of very low to low. Consequently, the observed improvements should be interpreted with caution and cannot be attributed solely to the educational interventions.

Despite these limitations, the findings support the potential value of continuing professional education in strengthening OC screening competence. Future well‐designed controlled studies using standardised outcome measures and longer follow‐up are needed to establish the effectiveness and sustainability of these interventions.

## Author Contributions

**Marina Sulyman**: conceptualisation, data curation, formal analysis, investigation, methodology, visualisation, writing – original draft. **Elizabeth Fitriana Sari**: conceptualisation, data curation, formal analysis, investigation, methodology, project administration, supervision, validation, visualisation, writing – review and editing. **Jyothi Tadakamadla**: supervision, validation, writing – review and editing. **Mariam Demian**: supervision, writing – review and editing.

## Funding

The authors received no specific funding for this work. Open access publishing facilitated by La Trobe University, as part of the Wiley ‐ La Trobe University agreement via the Council of Australasian University Librarians.

## Disclosure

All authors meet the authorship criteria of the International Committee of Medical Journal Editors (ICMJE). All authors have read and approved the final version of the manuscript and agree to be accountable for all aspects of the work, ensuring that any questions related to the accuracy or integrity of any part of the work are appropriately investigated and resolved.

## Ethics Statement

As this study is a systematic review based exclusively on published literature, ethical approval was not required.

## Conflicts of Interest

The authors declare no conflicts of interest.

## Supporting Information

Additional supporting information can be found online in the Supporting Information section.

## Supporting information


**Supporting Information** Table S1: Complete Database Search Strategy.

## Data Availability

Data sharing is not applicable to this article as no datasets were generated or analysed during the current study.
