# Supplementary material for: Effectiveness of Continuing Education Interventions in Improving Dentists’ Proficiency in Oral Cancer Screening: A Systematic Review and Quality Appraisal
Source: Int J Dent. 2026 Oct 7;2026:8479848. doi: 10.1155/ijod/8479848 (PMC13646708; doi:10.1155/ijod/8479848)
Supplement: Supplementary file 1 [file IJOD-2026-8479848-s001.docx]

**Supplementary Table S1. Complete Database Search Strategy**

| **Database** | **Search Strategy** |
| --- | --- |
| **PubMed** | ("Dentists"[MeSH] OR dentist* OR "dental practitioner*" OR "oral health professional*") AND ("Mouth Neoplasms"[MeSH] OR "oral cancer" OR "mouth cancer" OR "oral squamous cell carcinoma" OR OSCC) AND ("Education, Continuing, Dental"[MeSH] OR "Education, continuing"[MeSH] OR "continuing education" OR training OR "continuing professional development" OR "online learning" OR "digital education" OR "web-based learning" OR "distance learning") AND (diagnos* OR skill*, OR “screen*” OR knowledge OR improve*) |
| **Medline (OVID)** | (exp Dentists/ OR dentist*.mp. OR dental practitioner*.mp.) AND (exp Mouth Neoplasms/ OR oral cancer.mp. OR mouth cancer.mp. OR oral squamous cell carcinoma.mp. OR OSCC.mp.) AND (exp Education, Continuing/ OR exp Education, Continuing, Dental/ OR continuing education.mp. OR training.mp. OR continuing professional development.mp. OR online learning.mp. OR digital education.mp.) AND (diagnos* OR skill*, OR screen* OR knowledge OR improve*) |
| **CINAHL** | (MH "Dentists+") OR dentist* OR "dental practitioner*" AND (MH "Mouth Neoplasms+") OR "oral cancer" OR "mouth cancer" OR "oral squamous cell carcinoma" OR OSCC AND (MH "Education, Continuing+") OR "continuing education" OR training OR "continuing professional development" OR "online learning" OR "digital education" OR "web-based learning" AND (diagnos* OR skill*, OR “screen*” OR knowledge OR improve*) |
| **Google Scholar** | ("oral cancer" OR "mouth cancer" OR OSCC) AND (dentist* OR "dental practitioner") AND ("continuing education" OR training OR "continuing professional development" OR "online learning" OR "digital education") AND (diagnos* OR skill*, OR “screen*” OR knowledge OR improve*) |
